# High resolution radiation hybrid maps of bovine chromosomes 19 and 29: comparison with the bovine genome sequence assembly

**DOI:** 10.1186/1471-2164-8-310

**Published:** 2007-09-04

**Authors:** Aparna Prasad, Thomas Schiex, Stephanie McKay, Brenda Murdoch, Zhiquan Wang, James E Womack, Paul Stothard, Stephen S Moore

**Affiliations:** 1 Department of Agricultural, Food and Nutritional Science, University of Alberta, Edmonton T6G2P5, Alberta, Canada; 2 INRA, UR 875, Toulouse, France; 3 Texas A & M University, Texas, USA

## Abstract

**Background:**

High resolution radiation hybrid (RH) maps can facilitate genome sequence assembly by correctly ordering genes and genetic markers along chromosomes. The objective of the present study was to generate high resolution RH maps of bovine chromosomes 19 (BTA19) and 29 (BTA29), and compare them with the current 7.1X bovine genome sequence assembly (bovine build 3.1). We have chosen BTA19 and 29 as candidate chromosomes for mapping, since many Quantitative Trait Loci (QTL) for the traits of carcass merit and residual feed intake have been identified on these chromosomes.

**Results:**

We have constructed high resolution maps of BTA19 and BTA29 consisting of 555 and 253 Single Nucleotide Polymorphism (SNP) markers respectively using a 12,000 rad whole genome RH panel. With these markers, the RH map of BTA19 and BTA29 extended to 4591.4 cR and 2884.1 cR in length respectively. When aligned with the current bovine build 3.1, the order of markers on the RH map for BTA19 and 29 showed inconsistencies with respect to the genome assembly. Maps of both the chromosomes show that there is a significant internal rearrangement of the markers involving displacement, inversion and flips within the scaffolds with some scaffolds being misplaced in the genome assembly. We also constructed cattle-human comparative maps of these chromosomes which showed an overall agreement with the comparative maps published previously. However, minor discrepancies in the orientation of few homologous synteny blocks were observed.

**Conclusion:**

The high resolution maps of BTA19 (average 1 locus/139 kb) and BTA29 (average 1 locus/208 kb) presented in this study suggest that by the incorporation of RH mapping information, the current bovine genome sequence assembly can be significantly improved. Furthermore, these maps can serve as a potential resource for fine mapping QTL and identification of causative mutations underlying QTL for economically important traits.

## Background

Molecular genetic information of the major agricultural species, like cattle, is crucial in harnessing the benefit of genetic variation for economically important traits. The process of exploiting this information is greatly facilitated by the ordering of molecular markers along the chromosomes. High resolution RH mapping is a valuable approach to build maps, where both polymorphic as well as non-polymorphic markers can be included [1]. Of the several whole genome radiation hybrid panels available for cattle [2-5], the 12,000 rad whole genome RH (12K WG-RH) panel has been shown to have the highest mapping resolution [6-9]. Radiation hybrid maps also serve as one of the tools to facilitate the assembly of genome sequences [9-11]. Direct comparison of an RH map with a genome assembly allows identification of inconsistencies between the optimal marker order, found using the RH data, and the marker order observed in the current genome assembly.

The bovine genome sequencing project, started in 2003, has released three different assemblies of the genome. The first preliminary assembly (Bovine build 1.0), produced with 3X coverage, was released in September 2004; the second assembly (Bovine build 2.0) with 6.2X coverage in June 2005; and the third draft assembly (Bovine build 3.1) with 7.1X coverage in August 2006 [12]. The third draft assembly was produced using a combination of whole genome shotgun reads and BAC end sequences [12]. Previous comparisons of radiation hybrid mapping data with bovine genome sequence assembly (Bovine build 2.0) have shown large discrepancies on many chromosomes including BTA19 (156 mapped markers) and BTA29 (149 mapped markers) [10]. These discrepancies and the fact that there have been many QTL identified on these chromosomes [13-16], has prompted us to choose BTA19 and 29 as candidate chromosomes for high resolution mapping.

The traditional approach of RH mapping is to heuristically produce a so-called framework map, incorporating only a fraction of typed markers which are reliably ordered. However, a major disadvantage of building framework maps is that it positions the remaining unplaced markers into bins of confidence, which may not be of true order. Instead, we have constructed high resolution maps of BTA19 and 29 using the comparative RH mapping approach recently introduced in CarthaGène [17-19]. This approach is based on a probabilistic Bayesian model integrating the usual RH probabilistic model with a probabilistic model of breakpoint occurrences with a reference order, typically obtained from the position of orthologous markers in a related sequenced genome [20]. In this probabilistic model, breakpoints induced by chromosomal rearrangements are considered as rare events, following a Poisson law. Equivalently, we consider that genome assembly errors create rare spurious breakpoints between the RH map order and the current assembly order. Therefore, CarthaGène was used to produce a new RH map integrating the RH data with the current bovine genome assembly.

The objective of this study was to generate high resolution RH maps of BTA19 and 29, and to compare them with the current cattle genome sequence build. We also constructed cattle-human comparative maps of BTA19 and 29, which are known to be orthologous to human chromosome 17 (HSA17) and HSA11 respectively [21-23]. This comparative mapping information as well as the high resolution RH map provides an important independent source of information to improve the bovine genome sequence assembly.

## Results and discussion

### Genotyping of 12,000 rad panel and RH map construction

The bovine 12,000 rad panel was constructed to complement an existing 5000 rad panel and increase the mapping resolution [3,5]. We used SNP markers for RH mapping because of their availability from the bovine genome sequencing project, their abundance throughout the genome [24] and the ease and low cost of large scale SNP genotyping [25]. Correct SNP marker order is also essential for a variety of gene discovery approaches such as interval mapping or linkage disequilibrium based methods. The SNP markers were chosen from the bovine build 2.0 and typed on the 12 K WG-RH panel using the Illumina BeadStation Genotyping System [26]. This genotyping system produces reproducible and robust data due to its 30 fold redundancy at each locus. There is an average of 30 representatives of each bead type present on every array which allows for 30 independent genotypes of each SNP locus. Three positive (bovine genomic DNA) and three negative (rodent genomic DNA) controls were used in the experiment. All markers observed with even a small amount of amplification in any of the three negative controls were discarded. Also, any markers which did not exhibit clear cluster separation between positive and negative controls were discarded. The remaining markers were scored as described previously [27]. A total of 66.7% (668 out of 1001) loci on BTA19 and 68.4% (366 out of 535) loci on BTA29 were successfully amplified and scored. Markers were selected from the bovine build 2.0 which had a significant number of SNPs misassigned to the wrong chromosomes. Hence, out of 668 and 366 successfully amplified loci on BTA19 and 29, we mapped 555 and 253 markers on BTA19 and BTA29, respectively. The details of the SNP markers mapped on BTA19 and 29 are provided in Additional file 1. RH maps were constructed using the comparative mapping approach of CarthaGène software [17-19] which allows us to simultaneously exploit the RH data and the knowledge of a known related order. RH likelihood is sensitive to large scale ordering discrepancies, as produced by the assembly errors, but has difficulties to order closely related markers reliably. The assembly itself, despite possible assembly errors, is very informative at low scale (inside BACs). Because it exploits more data than pure RH mapping, it cannot be related to framework mapping. However, as shown earlier [20], integrating these two types of information produces high resolution maps of better quality. In this case, it also pinpoints likely assembly errors.

**Figure 1:**
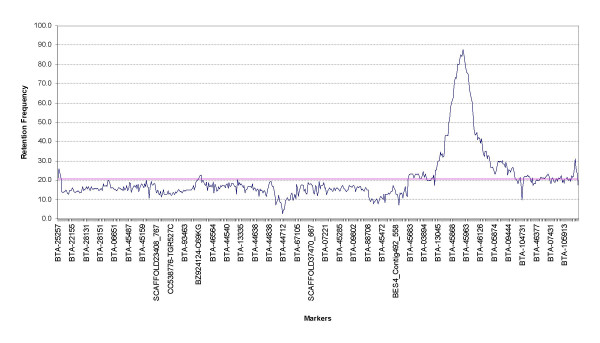
**Retention frequencies for 555 markers on BTA19**. Every seventeenth marker is shown on the X-axis and their corresponding retention frequencies on the Y-axis. The order of the markers in the graph corresponds to the order in the RH map. The left side of the horizontal axis represents centromere and right side represents telomere. The average retention frequency is shown by a pink coloured line in the chart.

**Figure 2:**
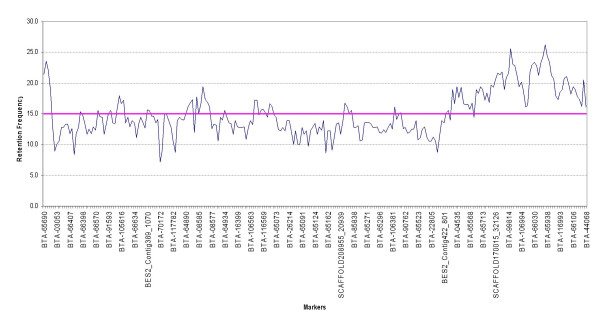
**Retention frequencies for 253 markers on BTA29**. Every sixth marker is shown on the X-axis and their corresponding retention frequencies on the Y-axis. The order of the markers in the graph corresponds to the order in the RH map. The left side of the horizontal axis represents centromere and right side represents telomere. The average retention frequency is shown by a pink coloured line in the chart.

On BTA19, we observed 455 different retention patterns, 390 unique retention patterns and 165 shared compatible retention patterns, out of 555 loci tested. The loci sharing compatible retention patterns suggest that they were so close that radiation could not induce any chromosomal break between them. The average retention frequency for all the mapped markers on BTA19 was 20.7% and varied from 2.8% for BTA-20935 to 87.7 % for BTA-45829 (Figure 1). The markers in the close vicinity of thymidine kinase gene on BTA19 reflected higher retention frequencies as this marker was used to select for hybrid cell lines [3]. Similarly on BTA29 we observed 215 different retention patterns, 193 unique retention patterns and 60 shared compatible retention patterns, out of 253 loci tested. The average retention frequency for all the mapped markers on BTA29 was 15.02% and varied from 7.2 % for BTA-70172 to 26.3% for BTA-09466 (Figure 2) with higher retention frequencies towards the telomeric end of the chromosome. Previous studies have reported that the pattern of retention frequencies varies markedly between chromosomes [2,9]. The total length of the RH maps of BTA19 and BTA29 extended to 4591.4 cR and 2884.1 cR, respectively [See Additional file 2]. Additional information about the maps, including the average resolution, and the range and standard deviation of the marker distances, is provided in Table 1.

**Table 1:** Summary statistics of the RH maps

**Statistics**	**BTA19**	**BTA29**
Markers typed on 12K RH Panel	1001	535
Markers successfully amplified	668	366
Markers mapped	555	253
Average retention frequency (%)	20.7	15.02
Markers with different retention patterns	455	215
Double markers	100	38
Total length (cR)	4591.4	2884.1
Bovine build 3.1 (bp)	63432577	44728515
Average resolution (Bovine build 3.1 (bp)/Markers with different retention patterns)	1 locus/139 kb	1 locus/208 kb
Range of marker distances (cR)	0.9–56.2	1.8–134.8
Standard Deviation	8.870832	16.214068

### Comparison with the bovine build 3.1 sequences

**Figure 3:**
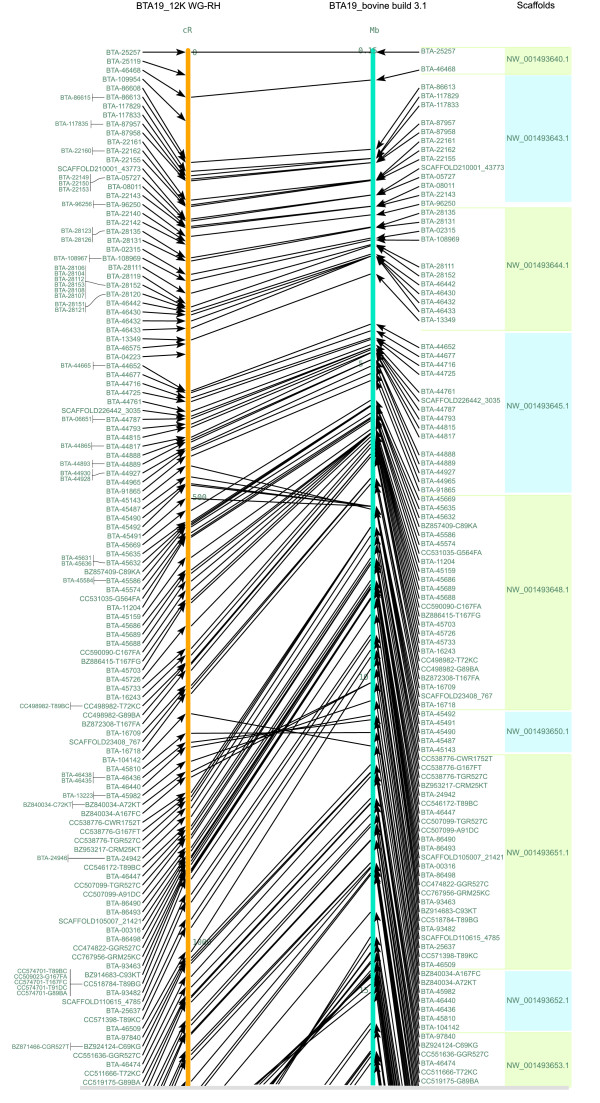
**RH map of BTA19 (left) compared with the corresponding bovine build 3.1 (right)**. This figure shows the upper quartile, for the full image please see Additional file 3. Lines between the maps connect markers in both maps. Distances of the RH map are scaled in (cR) CentiRays and on the bovine build 3.1 in (Mb) Mega base pairs. On the extreme right hand side, the coloured boxes represent scaffolds corresponding to each marker.

We aligned our chromosomal maps with the bovine build 3.1 sequences for BTA19 and BTA29 and found an overall agreement of order of loci between the two maps, although a number of inconsistencies were observed. Out of the 555 markers mapped to the 12K map of BTA19, 524 markers were assigned to BTA19 by the bovine genome sequence assembly. For 16 loci, we could detect scaffolds, which were not assigned to any chromosome by the sequence assembly [See Additional file 2, indicated in green colour]. Fourteen loci did not show acceptable hits with the bovine genome sequence assembly. One hundred and four markers were found to be incongruous and twelve scaffolds were found to be misplaced. Five scaffolds were found to be transposed and six were found to be inverted. In total, seventy four markers within scaffolds were found to be misplaced. One marker, BTA-29943, was assigned bovine chromosome 10 by the sequence assembly [See Additional file 2, indicated in yellow cells]. In addition, we observed a total of 8 gaps (more than 40 cR) on the BTA19 map (Figure 3 and Additional file 3).

**Figure 4:**
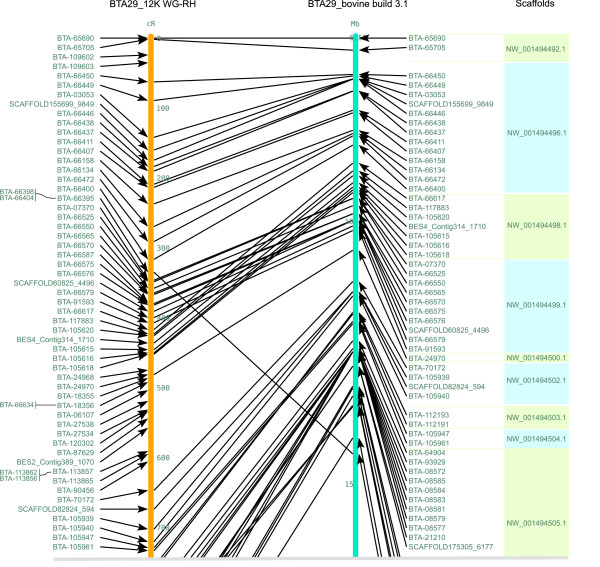
**RH map of BTA29 (left) compared with the corresponding bovine build 3.1 (right)**. This figure shows the upper quartile, for the full image please see Additional file 4. Lines between the maps connect markers in both maps. Distances of the RH map are scaled in (cR) CentiRays and on the bovine build 3.1 in (Mb) Mega base pairs. On the extreme right hand side, the coloured boxes represent scaffolds corresponding to each marker.

For BTA29, out of the 253 markers mapped, 215 markers were assigned to BTA29 by the bovine genome sequence assembly. Similarly, we could detect scaffolds for 25 loci, which were not assigned any chromosome by the sequence assembly [See Additional file 2, indicated in green colour]. Twelve loci did not show any acceptable hits with the sequence assembly. Forty five markers were found to be incongruous and ten scaffolds were found to be misplaced. Four scaffolds were found to be transposed and three scaffolds were found to be inverted. One marker, BTA-66150, was assigned bovine chromosome 15 by the sequence assembly [See Additional file 2, indicated in yellow cells]. In total, twenty five markers within scaffolds were found to be misplaced. Furthermore, we observed 5 gaps (more than 40 cR) on the BTA29 RH map (Figure 4 and Additional file 4).

For comparison, we computed the loglikelihood and length of maps built according to the bovine genome sequence order. We re-evaluated maps under a pure diploid RH model using all markers that had a match on the bovine build 3.1 sequences. There were 524 markers that were in common with bovine build 3.1 sequences and RH map of BTA19. The map built according to the bovine build 3.1 sequence order has a log-10-likelihood of -5000.69 and extends up to 6083.9 cR, whereas the map built according to our RH map order has a log-10-likelihood of -4303.72 and extends up to 4508.4 cR [See Additional files 5 and 6]. For BTA29, there were 215 markers that were common between RH map and bovine build 3.1 sequences. The map built according to the bovine build 3.1 sequence order has a log-10-likelihood of -2131.96 and extends up to 3822.5 cR, whereas the map built according to our RH map order has a log-10-likelihood of -1805.22 and extends up to 2763.7 cR [See Additional files 7 and 8]. Thus based on the RH data, the map derived from the bovine genome sequence is much less likely than our RH map order with log10-likelihood ratio differences of -696 and -326 for BTA19 and BTA29 respectively.

### Generation of the cattle-human comparative map

**Figure 5:**
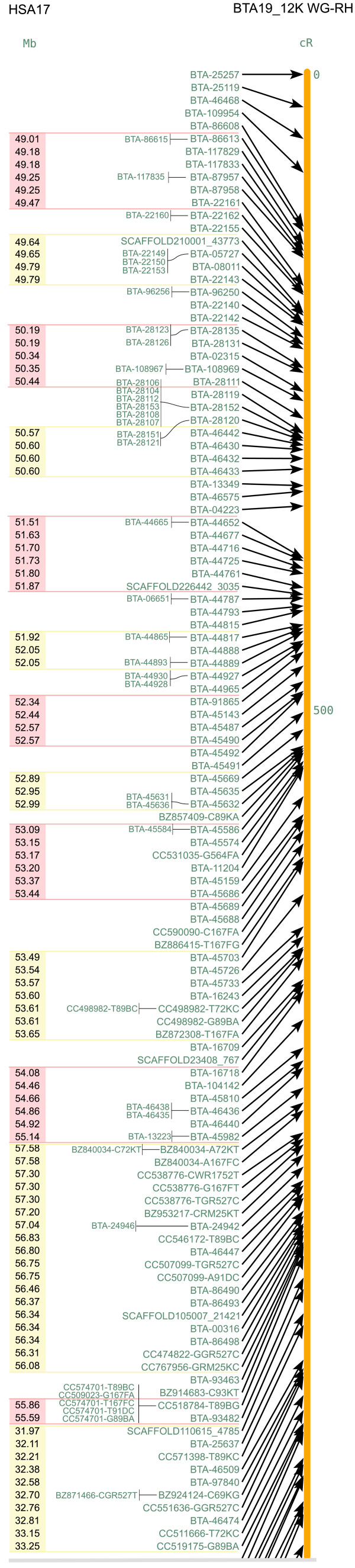
**Cattle-human comparative map of BTA19 (right) and HSA17 (left)**. This figure shows the upper quartile, for the full image please see Additional file 10. HSBs are coloured pink and yellow on HSA17 with the homologous sequence coordinates in the human genome (NCBI build 36) inside the HSBs.

**Figure 6:**
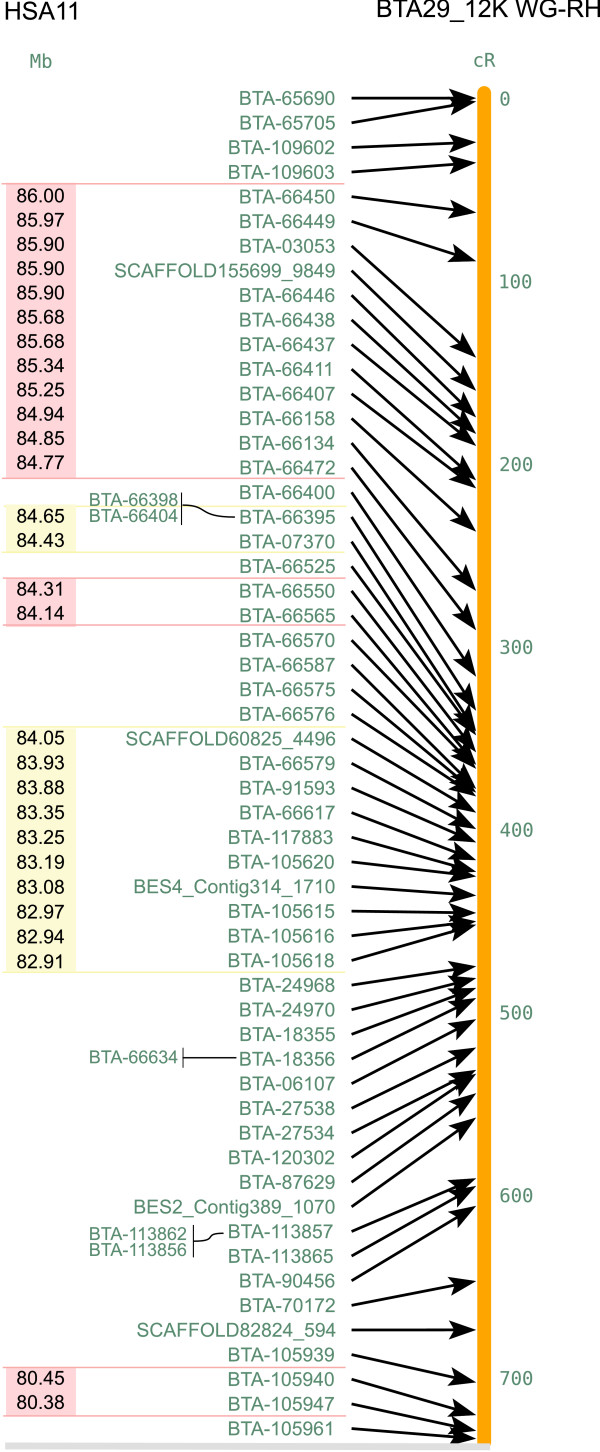
**Cattle-human comparative map of BTA29 (right) and HSA11 (left)**. This figure shows the upper quartile, for the full image please see Additional file 11. HSBs are coloured pink and yellow on HSA11 with the homologous sequence coordinates in the human genome (NCBI build 36) inside the HSBs.

Excluding binned markers, four hundred and fourteen (BTA19) and one hundred and seventy-five (BTA29) markers having human orthologs (reference assembly build 36 version 2) were used for the construction of cattle-human comparative maps. We identified 60 homologous synteny blocks (HSBs, ≥ 2 markers) on BTA19 and 23 HSBs on BTA29 as shown in Figures 5 and 6 respectively [See Additional files 9, 10, 11]. Also, 149 breakpoints were identified between BTA19 and the corresponding segments in the HSA17, while 51 breakpoints were identified between BTA29 and HSA11. We compared our maps with the previous studies [23,28]. The details of the number of markers used in all the three studies, number of HSBs, their size range and their median is provided in Table 2. The HSBs identified in our study are more in number as well as smaller in size because of the high density of markers mapped on the chromosomes. In addition, several of the 555 and 253 SNP markers mapped on BTA19 and 29 respectively, did not produce hits on the bovine (31 markers on BTA19 and 38 markers on BTA29) and human (50 markers on BTA19 and 45 markers on BTA29) chromosome sequences at the given expectation threshold, and some (10 markers on BTA19 and 6 markers on BTA29) produced hits on other human chromosomes, thus resulting in a larger number of smaller HSBs than previously described. The coordinates of our HSBs overall were in agreement with those identified in both earlier studies. However, small discrepancies in the orientation of a few HSBs were observed. Nine of the previously identified HSBs on HSA17 and 4 on HSA11 [28] were split into 60 and 23 HSBs respectively, in our study. In the Schibler et al. [23] study, 7 HSBs on HSA17 and 6 on HSA11 were split into 57 and 23 HSBs respectively. One of the HSBs on HSA17 (22.74–25.73 Mb) found in our study as well as in Everts-van der Wind et al. [28] study, was not reported by Schibler et al. [23]. The synteny block from 0.2–2.9 Mb identified in both of the previous studies [23,28] on HSA11 is absent from our comparative map. We have only 2 markers in that region and they both show hits in the human genome at the same position of 0.95 Mb. Therefore, although we cannot define them as a synteny block, our data supports the presence of the synteny block on HSA11. One region from 129–132 Mb in HSA11 shows disagreement across all the three studies and needs further investigation. The reason for minor discrepancies with the previous studies may be attributed to the use of different radiation hybrid panel and the mapping approach used.

**Table 2:** Comparison of the cattle-human comparative maps with previous studies

	**Prasad et al. 2007**		**Everts-van der wind et al. 2004 [28]**		**Schibler et al. 2006 [23]**	
	
	**BTA19**	**BTA29**	**BTA19**	**BTA29**	**BTA19**	**BTA29**
Total number of mapped markers	555	253	92	58	140	106
No. of HSB	60	23	9	5	7	7
Range of HSB sizes (Mb)	0.02–3.37	0.06–5.44	1.72–17.46	2.7–15.9	4.27–19.27	1.16–14.23
Median of HSB sizes (Mb)	0.44	0.44	5.29	8.5	10.56	4.35

## Conclusion

We have built a high resolution RH map of bovine chromosomes 19 and 29 consisting of 555 and 253 SNP markers, respectively. Maps of both the chromosomes, when compared with the bovine genome sequence assembly, show that there is significant internal rearrangement of the markers involving displacement, inversion and flips within the scaffolds and some scaffolds were found to be misplaced by the third draft (bovine build 3.1) of the bovine genome assembly. The RH maps reported here with an average resolution of 1 locus/139 kb and 1 locus/208 kb on BTA19 and BTA29 respectively, are useful for ordering SNP markers which can be used in future gene discovery investigations. Furthermore, they aid in the identification and rectification of potential errors in the current bovine genome sequence assembly.

## Methods

### Marker selection and genotyping of the RH panel

Sequence information for 1001 and 535 SNPs for BTA19 and BTA29, respectively, were obtained from public databases [29,30]. Out of 1001 SNPs, 68 SNPs were identified from the clones of CHORI-240 library spanning QTL regions for backfat reported previously [16,31]. Oligonucleotides respective to the markers were designed at the Bovine Genomics Laboratory at the University of Alberta and the oligo pooled assays (OPA) were synthesized and assembled by Illumina Inc. (San Diego, CA). The markers were genotyped on the 12,000 rad RH panel using the Illumina BeadStation 500G genotyping system [26]. Illumina GenCall Software was used to manually score the presence or absence of markers in 180 radiation hybrids as described previously [27].

### Statistical analysis of RH results

The RH maps of the chromosomes were constructed using the CarthaGène software [17-19]. Pairs of markers with compatible retention patterns (double markers) were identified and each pair was merged into one marker to simplify the search for an optimal map. Initially, the loglikelihood under the haploid equal retention model was used to find the best marker order as advocated in [32]. The bovine reference order files, which give the order of SNP markers in the bovine genome sequence assembly, were merged for the respective chromosomes using the *dsmergor *command. The traditional maximum multipoint likelihood criterion was replaced by the comparative mapping criterion using *dsbplambda *command, *lambda *set to 1. Then, the RH maps were built using the Lin-kernighan heuristic based commands: *lkh*, *lkhn*, *lkhl*, *lkhd*, *lkhocb *and *lkhocbn*. These commands are based on the 2-point based simplified model proposed in [33] or on LOD, distance and obligate chromosome breaks respectively. Parameters "1 0" were used to evaluate all maps encountered using the full probabilistic model. The best loglikelihood map found was then used as the starting point for the *greedy *command, which tries to improve maps using a taboo search algorithm. The map was further tested using a *flips *algorithm, which checks all possible permutations in a sliding window of fixed size (size 7 was used), and a *polish *algorithm, which checks the reliability of map by successfully removing one marker from the initial map and trying to insert in all possible intervals. Final maps distances were evaluated using the diploid equal retention model with an EM tolerance set to 10^-5 ^(using *cgtolerance*).

### Map comparison

Genomic sequence coordinates for SNPs were obtained by performing BLAST [34] comparisons between SNP flanking sequences and the bovine build 3.1 sequences, using an expectation value threshold of 1e-50. Most SNPs could be unambiguously placed on the genomic assembly using this method. Coordinates of the putative orthologous SNP regions in humans were obtained by performing BLAST searches against the latest human genome assembly (reference assembly build 36 version 2). Whenever possible, the SNP flanking sequence used in the human comparison was extended (up to 20,000 bp) using the bovine genome assembly, since the existing 500 bp flanking sequence did not produce a significant BLAST hit in most cases. An expectation value threshold of 0.00001 was used for comparison with the bovine and human genome sequence, and homologous synteny blocks (HSBs) were identified according to the criteria defined elsewhere [35]. The maps were drawn using the CarthaGène software [17-19].

## Authors' contributions

AP carried out genotyping, screened the RH panel, constructed chromosome maps, performed map and sequence comparisons and drafted the manuscript. TS built the maps, drafted the manuscript and provided intellectual contributions. SMK did data analysis. BM did genotyping of RH panel. ZW performed SNP selection and designed the Oligo Pool Assay. JEW developed and provided the 12,000 rad panel. PS placed marker sequences on the bovine and human genome assemblies. SM oversaw the genotyping and data analysis. All authors read and approved the final manuscript.

## Supplementary Material

Additional file 1 Sequence and IDs of SNP markers mapped on BTA19 and BTA29. Click here for file

Additional file 2 Map locations, bovine build 3.1, orthologous human and contig information for BTA19 and BTA29. The position of markers with compatible retention patterns are highlighted in blue colour. Empty cells represent no acceptable hits of the loci, when blasted with bovine and human genome sequence assembly. Cells shaded in yellow colour represent loci that were assigned chromosomes other than BTA19 and BTA29. The loci and their corresponding scaffolds which were unassigned by the bovine genome sequence assembly are indicated in green colour. Click here for file

Additional file 3 Full image of RH map of BTA19 compared with the corresponding bovine build 3.1 sequences. Click here for file

Additional file 4 Full image of RH map of BTA29 compared with the corresponding bovine build 3.1 sequences. Click here for file

Additional file 5 Log-likelihood and length of BTA19 map (524 markers) computed according to bovine build 3.1 genome sequence order. Click here for file

Additional file 6 Log-likelihood and length of BTA19 map (524 markers) computed according to our RH map order. Click here for file

Additional file 7 Log-likelihood and length of BTA29 map (215 markers) computed according to bovine build 3.1 genome sequence order. Click here for file

Additional file 8 Log-likelihood and length of BTA29 map (215 markers) computed according to our RH map order. Click here for file

Additional file 9 RH and human map coordinates for homologous synteny blocks for BTA19 and BTA29. Click here for file

Additional file 10 Full image of cattle-human comparative map of BTA19 and HSA17. Click here for file

Additional file 11 Full image of cattle-human comparative map of BTA29 and HSA11. Click here for file
