# Supplementary figures and images for: High resolution radiation hybrid maps of bovine chromosomes 19 and 29: comparison with the bovine genome sequence assembly
Source: BMC Genomics. 2007 Sep 4;8:310. doi: 10.1186/1471-2164-8-310 (PMC2064936; doi:10.1186/1471-2164-8-310)

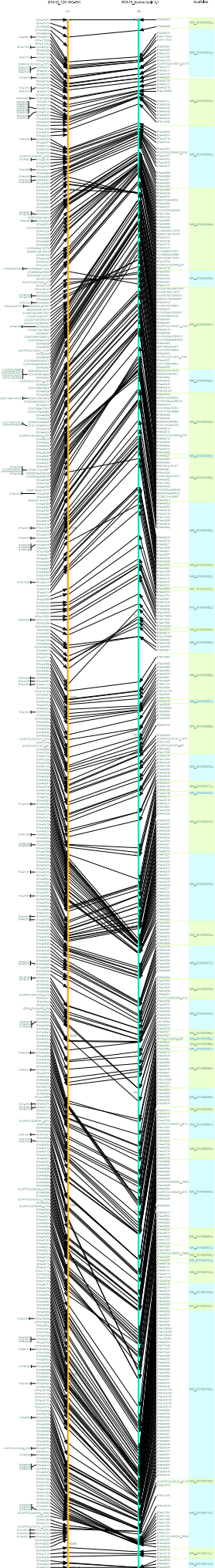

Supplement: Additional file 3 — Full image of RH map of BTA19 compared with the corresponding bovine build 3.1 sequences. [file 1471-2164-8-310-S3.pdf]

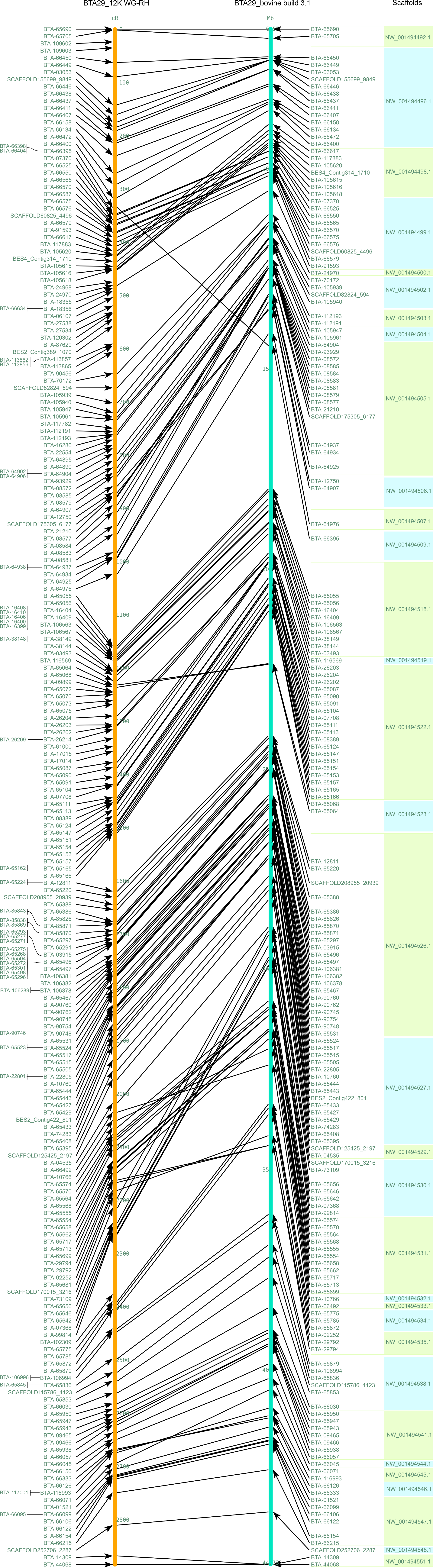

Supplement: Additional file 4 — Full image of RH map of BTA29 compared with the corresponding bovine build 3.1 sequences. [file 1471-2164-8-310-S4.pdf]

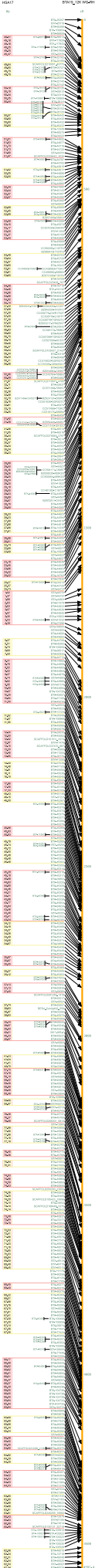

Supplement: Additional file 10 — Full image of cattle-human comparative map of BTA19 and HSA17. [file 1471-2164-8-310-S10.pdf]

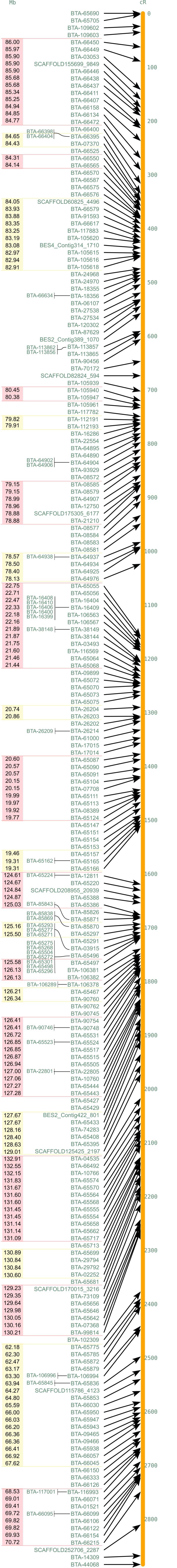

Supplement: Additional file 11 — Full image of cattle-human comparative map of BTA29 and HSA11. [file 1471-2164-8-310-S11.pdf]
